# SIDS-CDF hypothesis revisited: explaining hypoxia in SIDS

**DOI:** 10.1080/03009734.2016.1176972

**Published:** 2016-05-05

**Authors:** Pontus M. A. Siren

**Affiliations:** Singapore

**Figure 1.**
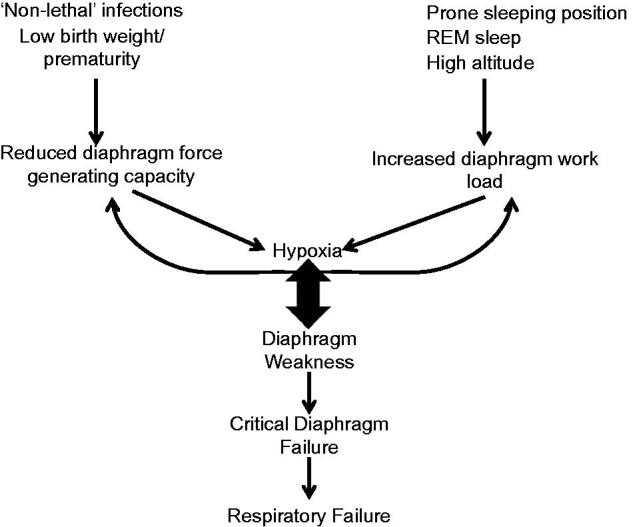
The SIDS-CDF hypothesis posits that several factors can affect the diaphragm force generating capacity and increase diaphragm workload resulting in hypoxia. Hypoxia can further reduce diaphragm force generating capacity and increase diaphragm workload. In some circumstances this can lead to diaphragm weakness, CDF and ultimately respiratory failure.

Dear Editor,

The sudden infant death syndrome (SIDS)–critical diaphragm failure (CDF) hypothesis (1,2) continues to attract attention 5 years after the publication of the original article in this journal, and several colleagues have contributed perspectives and insights to the hypothesis (3–6). The basic premise of the SIDS-CDF hypothesis is that the diaphragm is a vital organ that must continuously generate adequate force to maintain ventilation and that critical diaphragm failure is a terminal event and the cause of death in SIDS. Respiratory failure is a common cause of death of adults with compromised diaphragm function, and key SIDS factors have been shown either to reduce the diaphragm force-generating capacity or to increase the respiratory workload of the diaphragm. Both can cause hypoxia that can in turn further compromise diaphragm function and initiate a self-reinforcing feedback loop characterized by: weakened diaphragm – hypoxia – weakened diaphragm (Figure 1). Various types of non-lethal infections are commonly observed in SIDS victims, and a large body of research shows that diverse infections can cause a rapid and significant reduction in the force-generating capacity of the diaphragm. The prone sleeping position is another well-known risk factor for SIDS and one that has been especially difficult to explain in the context of SIDS etiology. However, clinical studies in infants show that in prone position the respiratory workload of the diaphragm is significantly increased. SIDS typically occurs during deep sleep when the supportive respiratory muscles are partially or totally inactivated, therewith increasing the respiratory load on the diaphragm. Gestational prematurity and low birth weight are risk factors for SIDS, and both affect the developmental and structural maturity and the force-generating capacity of the diaphragm, which achieves structural maturity after 6 months. The non-monotonic death rate in SIDS-CDF has been another enigmatic characteristic of the syndrome. The SIDS-CDF hypothesis posits that this is due to the passive immune protection provided by maternal antibodies during the first month of life when the respiratory muscles are most vulnerable. However, the passive immune protection quickly wanes 1 month *post partum*, especially in the absence of breast-feeding. A considerable amount of research has been conducted on disturbances in the serotonergic system of SIDS victims, and earlier we discussed how the SIDS-CDF hypothesis would explain the role of the tryptophan–serotonin–melatonin pathway in the etiology of SIDS (1). Altitude has also been shown to be an independent risk factor for SIDS (7) and is known to increase the respiratory workload on the diaphragm. These and other SIDS risk factors have been discussed in previous publications (1,2). However, I have not addressed in detail the role of hypoxia in SIDS, and since its significance has been highlighted in several recent publications, I will briefly review hypoxia in the context of the SIDS-CDF hypothesis and propose new research avenues and approaches.

Several studies over the past decades have highlighted hypoxia as a potentially central event in the etiology of SIDS (8,9). Indeed, already in the 1960s researchers suggested a link between SIDS and hypoxia (10). Recently, Neary and Breckenridge published a review article titled: ‘Hypoxia at the heart of sudden infant death syndrome?’ (11). While I agree with the observation that hypoxia plays a central role in SIDS, I will argue that SIDS is not a cardiac phenomenon, as Neary and Breckenridge suggest, but rather a respiratory event caused by the failure of the vital respiratory pump.

Hypoxia produces reliable biochemical markers that have been measured in SIDS victims and that suggest a prolonged period of hypoxia before death (8). A particularly compelling study investigated the correlation of SIDS and the vascular endothelial growth factor (VEGF) gene that is highly sensitive to changes in tissue partial oxygen tension. Evidence presented by Jones and colleagues suggests that SIDS victims experienced one or more hypoxic events over several hours to days before death, based on the time needed for gene up-regulation and subsequent protein expression and release to body fluids (12). What makes the VEGF gene study so compelling is that VEGF protein up-regulation has been previously demonstrated in experimental models, where animals were subjected to environmental hypoxia or to a variety of pathologic conditions with compromised tissue oxygenation. Following the publication of the VEGF study, Saugstad and Rognum emphasized that SIDS is preceded by hypoxia and urged researchers to: ‘intensify research identifying possible triggering factors inducing hypoxia and the cascade of events leading to SIDS’ (13). This communication is in part an attempt to address their unanswered 2003 question.

The respiratory system consists of two parts: a gas-exchanging organ, the lungs, and a pump that ventilates the lungs. The pump is composed of the chest wall, the respiratory muscles, the respiratory centers of the CNS, and the nerves connecting the centers to the muscles. CNS control of respiration and possible mechanical defects in the respiratory system have been extensively investigated in the context of SIDS (14,15). However, as we note in our original article, the possibility that SIDS is caused by critical weakness of the primary respiratory muscle has received very little attention, and as of March 2016, out of approximately 11,000 SIDS articles in PubMed, only 50 articles contained the search words ‘SIDS and diaphragm’, and only a few of those actually address diaphragm weakness in SIDS.

Fortunately, the diaphragm has been extensively investigated outside SIDS research, and its dysfunctions and vulnerabilities are well known. The diaphragm is the primary respiratory muscle, and it is axiomatic that the diaphragm powers the respiratory pump that oxygenates all muscles in the body, including the diaphragm itself. If the diaphragm becomes sufficiently weakened, respiration cannot be maintained (16). Acute respiratory failure due to critical diaphragm fatigue is common in patients with neuromuscular diseases and in some elderly patients (17,18). There is a large body of experimental evidence that shows that hypoxia exacerbates diaphragm fatigability and can impair the diaphragm’s ability to generate force. Mouse models have shown that acute hypoxic stress results in significant diaphragm muscle weakness (19), and in pigs acute hypoxemia impairs the diaphragm’s capacity to generate force (20). Brotto and colleagues note that hypoxia ‘has long been known to dramatically reduce muscle function’ (21), and in human adults it has been shown that hypoxia exacerbates both diaphragm and abdominal muscle fatigability and can contribute to respiratory failure (22,23). The characteristics of the failure of the ventilation pump are hypoxemia, hypoventilation, and hypercapnia (24). Patients who suffer from respiratory failure display increased work of breathing, muscle fatigue, and ventilation failure through imbalance between demand and supply (19). White and colleagues note that ‘it is well recognized that patients with respiratory muscle weakness are at risk of hypoxemia and hypercapnia during sleep, especially rapid eye movement (REM) sleep … Patients with respiratory muscle weakness show nocturnal hypoventilation, with oxygen desaturation particularly during rapid eye movement (REM) sleep … We conclude that *in patients with muscle weakness nocturnal oxygenation correlates with diaphragmatic strength*’ (emphasis mine) (25).

As we discussed in our original article in this journal, the young infant has a potentially vulnerable diaphragm. The diaphragm is largely inert during the gestational period and is structurally undeveloped. It is only approximately 2 mm thick, the supportive muscles are undeveloped, and in the prone sleeping position efficiency of the respiratory pump is reduced. Indeed, the prone position has been shown to increase hypoxia in infants, with a nadir in oxygenation coinciding with months 2–3 of life that are also the highest-risk periods for SIDS (26,27). Muller and colleagues go as far as to argue that ‘the normal preterm and term infant is very close to the threshold of diaphragmatic fatigue’ (28).

It is well known that that diaphragm weakness can cause acute respiratory failure in adults. The evidence discussed here suggests that SIDS victims experience prolonged and progressive hypoxia. Furthermore, it is known that key SIDS factors, including hypoxia, can both reduce the ability of the diaphragm to generate force and increase the respiratory workload. The SIDS-CDF hypothesis posits that the weakened diaphragm – hypoxia – weakened diaphragm self-reinforcing feedback loop could compromise ventilation and lead to respiratory failure.

This line of investigation offers promising new avenues for research, and the extensive body of research regarding the diaphragm can and should be leveraged in SIDS research. A specific area of research that could be fruitful is determining what kind of markers hypoxia or hypercapnia lasting several hours (perhaps even days) would leave on the diaphragm of a young infant and then determining if these markers can be found in SIDS victims. Fiber distribution in the diaphragms of SIDS victims has been regularly investigated, and no abnormalities have been found (personal communication with Professor Rognum). However, another potential marker to consider is the cross-sectional area of fibers, rather than fiber distribution per se (personal communication Professor O’Halloran). There are a number of other biomarkers for hypoxia that could be considered (lactate, ammonia, hypoxanthine, xanthine, and urate levels), and indeed Stoltenberg and colleagues have shown that SIDS victims have increased hypoxanthine concentrations in their vitreous humor (29). However, we must consider the possibility that critical diaphragm failure, which is essentially severe muscle weakness, does not leave *definitive* and easily measured markers in the muscle tissue of SIDS victims. Even if more accurate biomarkers can be established, the real challenge is not showing that SIDS victims suffer from hypoxia (this has already been established), but rather developing a model to test if a ‘weakened diaphragm–hypoxia–weakened diaphragm’ feedback loop can be caused by a combination of SIDS risk factors (infections, prone sleeping position, REM sleep, prematurity, absence of breastfeeding, cigarette smoke, altitude, etc.) and if this loop can cause the diaphragm to weaken to such an extent that it can no longer sustain respiration. I hope I have presented enough evidence here to encourage researchers to leverage the existing knowledge of the diaphragm muscle and its dysfunctions and to investigate further this novel and largely unexplored pathway for SIDS.

Finally, we should consider if the term *sudden* is not misleading in the context of SIDS. It seems that early practitioners sought to express the fact that the deaths were unexpected rather than rapid. Evidence reviewed here strongly suggests that SIDS is in fact characterized by a prolonged struggle to breathe, progressive hypoxia, and compromised tissue oxygenation. There is nothing sudden about SIDS.
